# Pulmonary Recovery Following Corrective Surgery in Adult Patients With Severe Scoliosis: A Minimum of Five-Year Follow-Up

**DOI:** 10.3389/fmed.2022.915904

**Published:** 2022-06-16

**Authors:** Xin Zhou, Zheng Zhang, Yue Yang, Jun Ma, Yichen Meng, Ce Wang, Xuhui Zhou

**Affiliations:** Department of Orthopedics, Second Affiliated Hospital, Naval Medical University, Shanghai, China

**Keywords:** pulmonary function test, halo gravity traction, severe scoliosis, lung volume, computed tomography

## Abstract

**Background:**

Halo gravity traction (HGT) has been reported to be a safe and effective adjunctive method for the management of scoliosis. However, the direct effects of HGT on the lung recovery of adult patients with scoliosis remain obscure.

**Objective:**

To investigate changes in lung volume and pulmonary function in adult patients with severe scoliosis who underwent posterior spinal fusion concomitant with preoperative halo gravity traction.

**Methods:**

A total of 47 patients with a minimum 5-year follow-up who underwent posterior spinal instrumentation and fusion using preoperative halo–gravity traction were analyzed. Pulmonary function tests and three-dimensional CT were performed to evaluate changes in lung function and lung volume, respectively.

**Results:**

There was significant change in the Cobb angle of the major curve after halo gravity traction (*P* < 0.0001). Significant improvement in both Cobb angle (*P* < 0.0001) and thoracic kyphosis (*P* = 0.034) after corrective surgery was observed. Pulmonary function did not change significantly during traction. However, a significant decline in absolute and percent-predicted pulmonary function values was noted following surgery. The average change in lung volume did not show statistical differences during traction. At 5-year postoperative follow-up, the mean values revealed a significant increase in total lung volume (*P* < 0.0001) and concave lung volume (*P* < 0.0001) with surgical correction, but no statistically significant change in lung volume on the convex side (*P* = 0.57). Postoperative pulmonary complications occurred in nine cases with lower preoperative pulmonary function, indicating the importance of performing spirometry before corrective surgery.

**Conclusions:**

We found that halo gravity traction prior to corrective surgery was less useful in improving pulmonary function in adult patients with severe scoliosis. However, these patients were expected to have increased lung volume after correction of the deformity.

## Introduction

Scoliosis is a complex three-dimensional spinal deformity characterized by a lateral spinal curvature with a Cobb angle > 10° and vertebral rotation. In addition to appearance concerns and neurological symptoms, severe curves over 90° can lead to cardiopulmonary insufficiency. The alveoli in the pulmonary tree do not fully develop until 8 years of age (1). Although spinal development during this period is closely related to lung growth. Spinal deformity limits the space available for lung growth and therefore affects lung compliance and lung volumes. Thus, a severe degree of thoracic scoliosis ultimately has detrimental effects on pulmonary function (2).

Correction of severe scoliosis remains a surgical challenge due to compromised cardiopulmonary function and the high risk of neurological impingement. Halo gravity traction (HGT) has been reported to be a safe and effective adjunctive method to reduce the severity of scoliosis and improve pulmonary function before correction of scoliosis (3, 4). However, the direct effects of HGT and corrective surgery on pulmonary recovery remain obscure. Some studies reported an insignificant decline in the pulmonary function test (PFT) (5, 6) while others describe significant improvements (7, 8). A possible reason for this difference was the poor ability of PFTs to quantify the effect of corrective scoliosis surgery on lung function. Brown et al. (9) and Dougherty et al. (10) have shown that lung volume can be accurately measured in patients with scoliosis using computed tomographic (CT) scan. Furthermore, most published studies have focused on long-term pulmonary function in young patients (<18 years of age) with scoliosis (11–14). There was still a scarcity of data on adult patients with severe scoliosis. Thus, the goal of this study was to measure changes in lung volume and pulmonary function in adult patients with severe scoliosis who underwent posterior spinal fusion concomitant with preoperative HGT.

## Materials and Methods

Patients who underwent correction surgery from January 2012 through December 2016 at our institution and met the inclusion criteria were selected. Inclusion criteria were as follows: (1) adult patients (over 18 years) diagnosed with scoliosis, with a major thoracic curve > 90 degree; (2) undergoing operations with preliminary HGT; and (3) had a complete record of demographic, preoperative and postoperative pulmonary CT radiographic and pulmonary function data. Patients with kyphosis-dominant deformities or any congenital cardiopulmonary disease were excluded from this study. This study was approved by the Ethics Committee of the Second Affiliated Hospital of Naval Medical University and the written informed consent of the patients was obtained before treatment.

Radiographs, lung volume assessment and PFTs were conducted before traction, and were repeated preoperatively, and then 5 years postoperatively. For lung volume assessment, CT scans in accordance of ALARA (as low as reasonably accurate) protocols were obtained on a 64-slice scanner (GE Light Speed VCT, GE Healthcare, Chicago, IL, USA). Patients were in supine position and instructed to hold their breath in deep inspiration during CT scanning. Technical parameters were: beam current 52 to 185 mA, tube potential 120 kV, slice collimation 5 mm; slice thickness 5 mm and pitch 1.375. The ALARA (as low as reasonably accurate) protocols were also used to limit the radiation dose. The raw data obtained were reformatted on the Aquarius Workstation v4.4 (TeraRecon, Inc., San Mateo, CA, USA), followed by 3D volumetric reconstructions of the lung parenchyma with threshold limits of −1024 to −200 Hounsfield units using TeraRecon software. Total lung volume, convex side lung volume, concave side lung volume were measured by an experienced radiologist. CT scan is not routinely performed on scoliosis patients during their follow-up due to cost and ionizing radiation exposure. Patients in the present study undergone CT scan prior to traction and after traction to assess the structures of the vertebrae and vertebral pedicles, and at 5 years follow-up to evaluate bony fusion and instrumentations. Parameters describing coronal and sagittal deformity measured on radiographs included: Magnitude of main thoracic curves and minor curves; thoracic kyphosis (T5–12); curve flexibility on supine bending radiograph and radiograph performed with the patient in HGT, and the final radiograph taken before surgery was used for calculating the HGT flexibility; sagittal balance, measured as the horizontal distance between the C7 plumb line (C7PL) and the posterior-superior corner of the S1 vertebral body, and defined as negative if the C7PL was posterior to the S1 posterior-superior corner; coronal balance, defined as the distance between the center sacral vertical line (CSVL) and the C7PL. The coronal balance was defined as negative when the C7PL was to the left side of the CSVL. Spirometry was assessed using Jaeger MasterScreen PFT System (Erich Jaeger GmbH, Würzburg, Germany) by measuring the predicted forced vital capacity as a percentage of predicted normal value (FVC%) and the ratio of forced expiratory volume in 1s/FVC (FEV1%). These two parameters has been proved to provide an adequate assessment of function in terms of volume and flow as measured by the pulmonary function tests (15). The patient's arm span was used as representative of height. Patients were classified as having improved if their FVC% improved by at least 5% and as stable if there was an improvement or decrease of 0–5%; worsen if the FVC% dropped more than 5% after treatment (16). Pulmonary impairment was categorized using American Thoracic Society guidelines for percent predicted values: >80% normal, ≤80 to >65% mild, ≤65 to ≥50% moderate, < 50% severe impairment (17).

The HGT strategy has been reported elsewhere (3). Traction was usually started immediately with a low weight of 1.5–2.5 kg. Traction was gradually increased at a rate of 1.0–1.5 kg per day as tolerated. The target weight was to reach a maximum traction of 33–50% of the patient's body weight. Traction was applied while in a wheelchair for a minimum of 10 h per day. The patients had daily neurologic examinations and were encouraged to take breathing strengthening exercises. HGT-related complications were recorded. HGT was halted immediately if a neurologic deficit appeared.

All data were expressed as means, standard deviations and ranges. Statistical analysis was performed using SPSS 24.0 software (SPSS Inc., Chicago, IL, USA). The differences in radiological parameters, pulmonary function and lung volume between different time points were compared using repeated measures ANOVA. Fisher's exact test or the chi square test was used for categorical variables. Student's *t-*tests were used to test for significant differences. Pearson's correlations were used to assess the association between radiographic measurements and spirometric test and lung volume results. A two-tailed *P*-value < 0.05 was considered to be statistically significant.

## Results

### Demographics

From January 2012 to December 2016, a total of 47 patients (16 males and 31 females) with severe scoliosis were enrolled, with a mean age of 29.5 years (range, 19–41) and follow-up of 5.4 years (range, 5.1–5.7 years). Etiological diagnoses were neuromuscular (*n* = 13), idiopathic (*n* = 22) and congenital (*n* = 12) scoliosis. All 47 curves were major thoracic/thoracolumbar with apex in between T7 and T10 vertebra. Upper end vertebra of major curve varied from T2 to 6 and lower end vertebra varied from T11 to L3. All patients were treated with one-stage posterior approach alone. The average number of fused segments was 14.9 (range, 13–17). Osteotomies were performed in 39 patients (in 18 patients, pedicle subtraction osteotomy alone; in nine patients, vertebral column resection alone; in seven, Smith-Petersen osteotomy, as well as pedicle subtraction osteotomy; and in five, Smith-Petersen osteotomy, as well as vertebral column resection). The average traction period for all patients was 74.9 days (range, 33–125 days) and average maximum traction force was 20.9 kg (range, 10–25 kg). There was one case of pin-site infection requiring debridement. No neurologic deficit was observed during traction or surgery. The results were summarized in Table 1 and Supplementary Table 1.

**Table 1:** Characteristics of the patients included in the study.

**Parameters**	**Values**
Age (yr)	29.5 (5.8)
Range	19–41
Weight (kg)	47.3 (9.9)
Height (cm)	156.1 (51.4)
Diagnosis	
Idiopathic scoliosis	22
Congenital scoliosis	12
Neuromuscular scoliosis	13
Follow-up (yr)	
Mean	5.4
Range	5.1–5.7
Sex (male/female)	16/31
Halo gravity traction duration (day)	
Mean	74.9 (25.4)
Range	33–125
Maximum traction force (kg)	
Mean	20.9 (4.5)
Range	10–25
Intraoperative blood loss (ml)	1,511.5 (996.5)

### Radiographic Evaluations

All curves were rigid with an average flexibility of 11.8% (range, 1.9–24.7%), which was not significantly different from the HGT flexibility (12.5%, *p* = 0.38). The average Cobb angle of major curve was corrected from 99.5° ± 10.2° (range, 90.3–131.4°) before traction to 87.3 ± 12.5° (range, 67.8–120.1°) before ultimate corrective surgery (*P* < 0.0001). The kyphosis pre-traction was 47.5 ± 40.4° and measured 42.9 ± 34.3° on the HGT radiographs, representing a significant difference (*p* = 0.0004). The coronal and sagittal balance prior to halo was 5.2 ± 21.5 mm and 1.9 ± 22.1 mm, respectively. After traction, the coronal balance was 23.7 ± 26.3 mm (<0.0001) and the sagittal balance was 6.3 ± 25.7 mm (*P* = 0.59). After surgery, the average Cobb angle was44.6 ± 14.5° (correction rate 48.9%). No significant loss of correction at the last follow up (*P* = 0.085). The average thoracic kyphosis was corrected to 34.8°. There was a significant improvement in both Cobb angle (*P* < 0.0001) and major kyphosis (*P* = 0.034) following surgery. The post-operative coronal balance was significantly different relative to the post-traction coronal balance (*P* = 0.0002) but similar to the pre-traction value (*P* = 0.068). Sagittal balance was not significantly changed from post-traction to post-operation (*P* = 0.24), and remained at 5.6 mm at 5 years follow-up (*P* > 0.05). Radiographic changes are shown in Table 2.

**Table 2:** Radiographic results.

**Outcome**	**Pre-traction**	**Post-traction**	**Post-operation**	**Postoperative 2-year follow-up**	**Postoperative 5-year follow-up**
Major curve (°)	99.5 (10.2)^†^^§^^¶^^‡^	87.3 (12.5)*^§¶‡^	44.6 (14.5)*^†^	44.7 (14.2)*^†^	45.3 (14.3)*^†^
Thoracic kyphosis (°)	47.5 (40.4)^†^^§^^¶^^‡^	42.9 (34.3)*^§^^¶^^‡^	34.8 (10.4)*^†^	35.0 (10.1)*^†^	37.3 (11.1)*^†^
Coronal balance (mm)	5.2 (21.5)^†^	23.7 (26.3)*^§^^¶^^‡^	4.0 (25.9)^†^	6.8 (22.0)^†^	6.7 (22.3)^†^
Sagittal balance (mm)	1.9 (22.1)^†^	6.3 (25.7)*^§^^¶^^‡^	0.2 (20.6)^†^^‡^	2.6 (20.4)^†^	5.6 (23.3)^†^^§^
PI (°)	49.7 (15.4)	48.8 (15.5)	49.3 (15.1)	49.4 (15.3)	49.3 (15.0)
PT (°)	11.4 (17.2)^†^	6.6 (13.7)*^§^^¶^^‡^	10.6 (12.8)^†^	10.9 (13.2)^†^	10.9 (13.0)^†^
SS (°)	38.7 (16.8)^†^	43.6 (16.2)*	37.5 (12.6)	38.1 (12.4)	38.0 (12.6)
LL (°)	−68.3 (24.1)	−67.9 (27.6)	−56.5 (14.9)	−57.0 (15.2)	−57.1 (15.3)

*PI pelvic incidence, PT pelvic tilt, SS sacral slope, LL lumbar lordosis.*

*Values are expressed as mean (SD).*

* *Statistically significant compared with values pre-traction.*

† *Statistically significant compared with values post-traction.*

§ *Statistically significant compared with values post-operation.*

¶ *Statistically significant compared with values at postoperative 2-year follow-up.*

‡ *Statistically significant compared with values at postoperative 5-year follow-up.*

*P < 0.05 was taken as statistically significant*.

### Spirometric Results

All patients had complete longitudinal data with spirometric tests performed prior to traction, after traction, after surgery, and at 2 years and 5 years follow-up, and comparisons of spirometry in terms of FVC, FVC%, FEV1 and FEV1% changes were made between different time points (Table 3). Absolute and percent-predicted values FVC (FVC%) and FEV1(FEV1%) values were 2.31 ± 0.81 (62.3 ± 16.8) and 1.97 ± 0.73 (64.9 ± 14.3), respectively, before traction. Neither FVC% (*P* = 0.41) nor FEV1% (*P* = 0.19) correlated with the Cobb angle. FVC and FEV1 values at the time of pre and post traction showed no statistical difference (*P* = 0.38 and *P* = 0.96, respectively). Both FVC% and FEV1% post-traction were higher than the pre-traction values; however, the differences were not statistically significant (*P* = 0.34 and *P* = 0.37, respectively). After operation, absolute FVC and FEV1 values decreased an average of 0.42 L (*P* = 0.0013) and 0.30 L (*P* = 0.048), respectively. FEV1% declined significantly as well by 7.9% (*P* < 0.001). A clinically significant decline (a decline of ≥ 10% in FEV1%) was observed in 44%. FVC% declined by 3.2%, which was not significant (*P* = 0.29). FVC, FEV1 and FEV1% showed gradual rises between post-operation, 2 years follow-up, and 5 years follow-up, with significance between post-operation and 2 years follow-up, and post-operation and 5 years follow-up (*P* < 0.05). FVC% showed no significant change over the three time points (*P* > 0.05). Although all four spirometric variables have increased from start to the final follow-up, the differences were not significant. Prior to traction, 36.2% were normal, 42.6% had mild impairment, and 21.2% had moderate-severe impairment, while at 5 years post-operatively, 38.3% were normal, 48.9% had mild impairment, and 12.8% had moderate-severe impairment (*P* = 0.54). The FVC% improved > 5% in eight patients (17.0%). Thirty two patients (68.1%) remained stable, and 7 (14.9%) declined > 5%. There was no significant correlation between the amount of change in Cobb angle and the change in spirometric test: *r* = −0.26, *P* = 0.185 for FEV1%, and *r* = −0.35, *P* = 0.075 for FVC%.

**Table 3:** Spirometric results and lung volume results.

**Outcome**	**Pre-traction**	**Post-traction**	**Post-operation**	**Postoperative 2-year follow-up**	**Postoperative 5-year follow-up**
FVC (L)	2.31 (0.81)^§^	2.50 (0.98)^§^	2.08 (0.75)*^†^^¶^^‡^	2.45 (0.81)^§^	2.45 (0.83)^§^
FVC%	62.3 (16.8)	63.7 (13.9)	60.5 (14.2)	65.7 (10.7)	64.1 (15.2)
FEV1 (L)	1.97 (0.73)^§^	1.93 (0.80)^§^	1.63 (0.59)*^†^^¶^^‡^	2.17 (0.55)^§^	2.28 (0.70)^§^
FEV1%	64.9 (14.3)^§^	66.6 (18.2)^§^	58.7 (16.3)*^†^^¶^^‡^	68.5 (12.5)^§^	69.8 (11.4)^§^
Total lung volume	888.5 (19.9)	860.8 (36.9)^‡^	NA	NA	1095.9 (59.4)*^†^
Concave side lung volume	339.7 (21.3)	327.7 (42.6)^‡^	NA	NA	514.7 (46.3)*^†^
Convex side lung volume	548.8 (18.4)	533.1 (33.3)	NA	NA	581.2 (63.2)

*FEV1 forced expiratory volume in 1s, FEV1% the actually measured value/predicted value of FEV1, FVC forced vital capacity, FVC % the actually measured value/predicted value of FVC, NA, Not applicable.*

*Values are expressed as mean (SD).*

* *Statistically significant compared with values pre-traction.*

† *Statistically significant compared with values post-traction.*

§ *Statistically significant compared with values post-operation.*

¶ *Statistically significant compared with values at postoperative 2-year follow-up.*

‡ *Statistically significant compared with values at postoperative 5-year follow-up.*

*Not applicable.*

*P < 0.05 was taken as statistically significant*.

### Lung Volume Results

**Figure 1:**
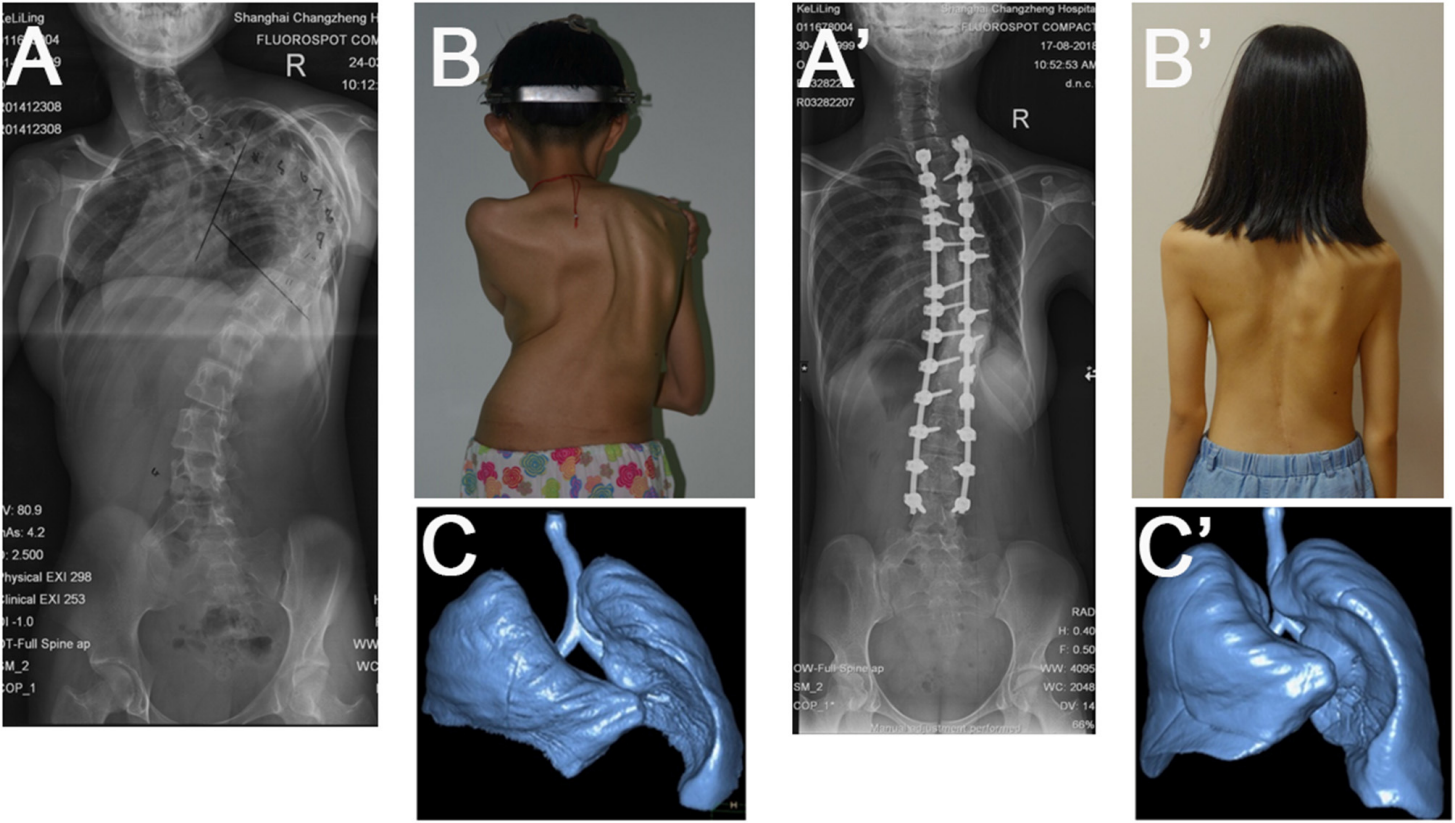
A 20-year-old female with early-onset idiopathic scoliosis. The initial Cobb angle was 112° **(A,B)**. Halo gravity traction and posterior correction with instrumentation were performed. At 5-year follow-up, the scoliosis had decreased to 45° **(A',B')**. Improvement in the lung volumes was noted when compare preoperative reconstructed computed tomographic scan of the lungs **(C)** with ones at the last follow-up **(C')**.

The patients were found to have mean values (cm^3^) of 888.5, 548.8, and 339.7 for total lung volume, convex side lung volume and concave side lung volume, respectively, before HGT (Table 3). After HGT, the mean values were 860.8, 533.1, and 327.7. The average change exhibited no statistical difference. At 5-year follow-up, the mean values were 1095.9, 581.2, and 514.7. This corresponded to average change of 235.1, 48.1, and 187.0 for total lung volume, convex side lung volume and concave side lung volume, respectively. Repeated measures ANOVA revealed significant increase in total lung volume (*P* < 0.0001) and concave side lung volume (*P* < 0.0001) with surgical correction, but no statistically significant change in convex side lung volume (*P* = 0.57). There was a statistically significant positive correlation between the Cobb angle change after deformity correction and the change in lung volume (*r* = −0.79, *P* = 0.005). However, the change in Cobb angle was not significantly correlated with changes in convex side lung volume change (*P* = 0.38) or concave side lung volume change (*P* = 0.26). In addition, we found a strong correlation between the percent change in FVC values and the percent change in the lung volume of the concave side (*r* = 0.98, *P* < 0.001), while we did not find this correlation on the convex side (*r* = 0.16, *P* = 0.44). A subgroup analysis demonstrated etiology of scoliosis had no impact on all four spirometric variables and lung volume changes (Supplementary Table 2). Typical case is shown in Figure 1.

Postoperative pulmonary complications included four cases of pleural effusion and five cases of mild pneumonia. Preoperative FVC% had a significant impact on the lung complications incidence (*P* = 0.031), which was lower preoperative pulmonary function values increased the risk of postoperative pulmonary complications.

## Discussion

The severe degree of scoliosis affects the size and dimension of the thoracic cage and, accordingly, the pulmonary function. After 90° of curvature, there is severe affection for lung volume and respiratory system compliance is decreased to levels comparable to adult respiratory distress syndrome (18). Pulmonary recovery changes have been evaluated in AIS patients, but never before in adult patients with severe scoliosis. Study of this population is more difficult than for population of adolescent idiopathic scoliosis because pulmonary reserves peak, in terms of age, in the mid - 20s and decline with increasing age (19, 20). In addition, most previous studies determined PFT using spirometers. While CT-based measurements circumvent many of the obstacles associated with obtaining accurate PFTs in children (21). We are the first to compare the change in lung volume using 3D CT in adult patients with scoliosis. The present study revealed that for adult patients with severe scoliosis, perioperative HGT showed no significant effects in improving lung volume or pulmonary function. While after corrective surgery, these patients had a significantly improved PFT results and increased lung volume.

Previous studies have demonstrated that perioperative HGT could promote pulmonary recovery, but the data were inconvincible. Swank reported 20 patients with severe scoliosis undergoing preoperative HGT and noted improvements in PFT results in some patients but a decline or no improvement in others (22). Nepple presented a case with severe thoracic scoliosis and respiratory insufficiency, and after 4 weeks of HGT the patient's FVC% improved from 31 to 47% (23). In the study of Koller et al. (16), HGT achieved a significant increase of 9% in FVC% was in patients with severe pulmonary impairment. Our study showed both FVC% and FEV1% improved slightly, but the difference has no statistical significance. Different curve correction results were also noted compared with that of the literature. In Sink's report (24), the Cobb angle of the major curve improved by 35% from an average of 84° to 55° before the fusion. Watanabe et al. (3) reported that preoperative halo–gravity traction allowed correction of the scoliotic deformity an average of 23.3%. In the present study, a mean of 12.3% from an average of 99.5° to 87.3° correction was achieved with HGT. The flexibility Huangpu of the HGT radiographs is supposed to increase compared to the radiographs on admission. However, the difference was not significant. In the present study, we also evaluated the change in lung volume using CT reconstruction. Our results showed that preoperative HGT did not change lung volume significantly. This may be attributed to the fact that all of the patients included in our study reached skeletal maturity and HGT could not induce a meaningful release effect on such rigid curves.

Several reports exist on the relationship between corrective surgery and changes in pulmonary function. Most data are derived from moderate idiopathic curves with no to mild pulmonary impairment and are conflicting. Generally, an approximate 5–10% increase of the PFT values was observed at 12 months after corrective surgery (25). A retrospective study conducted by Lehman revealed that adult patients with preoperative pulmonary impairment (FEV1 of < 65%) had an overall 2.7% improvement in pulmonary function at postoperative 2-year follow-up, which was significantly different from the decline in pulmonary function for all other patients (26). Therefore, we expected that the PFT results of our patients may be improved after correction and collected data to evaluate lung function at 2-year and 5-year follow-up postoperatively. However, the results demonstrated that, the lung function in three-fourth patients decreased slightly following surgery, but recovered to 3% higher than preoperative level at the last follow-up. This increase was not clinically significant. Similar results had also been observed in the study by Yuan. The PFT results decreased significantly immediately after surgery and returned to baseline by 2 months postoperatively (27). The change of lung volume showed the same trend as that of PFT. This result may be explained by the fact that patients in the present study were restricted complete activities for the first year postoperatively. And when they returned to full activities, pulmonary recovery would get further promoted. Several authors recommended the use of Cobb angle assessments for pulmonary function. On the contrary, our results did not show a correlation between Cobb angle and PFT. However, there was a strong correlation between the change in the Cobb angle before and after and the change in lung volumes. Furthermore, the percent change in the lung volume on the concave side showed a strong correlation to the percent change in the PFT results, while the convex side did not show this correlation. A previous 3D CT scan-based study demonstrated that the concave side lung is affected to a great extent compared to the convex side with increasing severity of scoliosis (28). The possible reason why the concave lung is more affected than the convex lung could be that there is less space available for the lung on the concave side resulting from longitudinal growth inhibition (29). The convex side lung may over-compensate for the restriction of the concave side lung. After surgery, the convex side lung did not improve significantly possibly due to a ceiling effect and the concave side lung volume improved significantly to balance the other side. Postoperative pulmonary complications occurred in 19% of the patients in our study. And we found that the occurrence of pulmonary complications was correlated with preoperative FVC%, which highlighted the importance of preoperative evaluation of pulmonary function.

There were some limitations in this study. First, there are no guidelines on when to measure lung volume on CT scan. It should be noted that routine use of CT scan is not recommended as a means of evaluating lung function secondary to concern for increased radiation and economic burden. The patients in our study received a CT scan at 5-year postoperative follow-up due to concerns about implants. And all scans were obtained using ALARA protocols. Second, the patients in our study did not have the same laterality of their curves. As a result, the presence of the heart may cause a disparity in the evaluation of lung volume. Third, the present study was of retrospective design with a small sample size, which limited the power of our results. Fourth, the CT scans in the present study were obtained under breath-hold conditions. The effects of tidal volume on lung volume as measured by CT scan reconstruction remains to be determined.

Our present study suggests that the role of HGT preceding corrective surgery is limited in improving pulmonary function in adult patients with severe scoliosis. However, these patients are expected to have increased lung volume after correction for the deformity. More large-scale prospective studies should be performed to further confirm our results in the future.

## Data Availability Statement

The original contributions presented in the study are included in the article/Supplementary Materials, further inquiries can be directed to the corresponding authors.

## Ethics Statement

The studies involving human participants were reviewed and approved by Ethics Committee of the Second Affiliated Hospital of Naval Medical University. The patients/participants provided their written informed consent to participate in this study.

## Author Contributions

XiZ, YM, and ZZ participated in study design, data analysis and interpretation, writing the manuscript, and critical revision of the manuscript. YY and JM were involved in data collection, preliminary data analysis and interpretation, critical revision of the manuscript, and statistical expertise. CW and XuZ were involved in study conception and design, data analysis and interpretation, writing the manuscript, critical revision of the manuscript, supervision, and administrative support. All authors contributed to the article and approved the submitted version.

## Conflict of Interest

The authors declare that the research was conducted in the absence of any commercial or financial relationships that could be construed as a potential conflict of interest.

## Publisher's Note

All claims expressed in this article are solely those of the authors and do not necessarily represent those of their affiliated organizations, or those of the publisher, the editors and the reviewers. Any product that may be evaluated in this article, or claim that may be made by its manufacturer, is not guaranteed or endorsed by the publisher.
