# Supplementary material for: Pulmonary Recovery Following Corrective Surgery in Adult Patients With Severe Scoliosis: A Minimum of Five-Year Follow-Up
Source: Front Med (Lausanne). 2022 Jun 16;9:915904. doi: 10.3389/fmed.2022.915904 (PMC9243643; doi:10.3389/fmed.2022.915904)
Supplement: Supplementary file 1 [file Table_1.DOCX]

| Supplementary table 1. Patient characteristics | | | | | | | | | | | | |
| --- | --- | --- | --- | --- | --- | --- | --- | --- | --- | --- | --- | --- |
| Case | Gender | Age (years) | BMI | Etiology | Coronal major curve (°)/Range/Apex | Flexibility index (%)* | Coronal balance (mm) | Thoracic kyphosis (°) | Sagittal balance (mm) | Traction duration (days)/ Maximum weight (kg) | Definitive fusion range | Osteotomy |
| 1 | F | 19 | 17.4 | IS | 94.3/T2-L1/T10 | 9.6 | 24.3 | -19.2 | -19.6 | 42/15 | T2-L4 | PSO |
| 2 | F | 27 | 18.6 | IS | 91.1/T3-T12/T8 | 7.8 | 36.7 | 72.3 | -27.5 | 51/15 | T1-L3 |  |
| 3 | F | 32 | 21.7 | IS | 131.4/T3-L1/T8 | 2.4 | 18.1 | 117.2 | -36.5 | 91/20 | T2-L2 | VCR |
| 4 | F | 35 | 14.8 | IS | 109.7/T5-L1/T10 | 16.7 | -28.5 | 63.5 | -31.3 | 56/10 | T1-L5 | VCR |
| 5 | F | 19 | 22.9 | IS | 98.4/T3-L2/T9 | 8.3 | 3.9 | 17.9 | 26.9 | 105/25 | T2-L3 |  |
| 6 | F | 25 | 15.5 | IS | 108.6/T6-L2/T10 | 9.3 | -14.4 | 42.7 | 8.6 | 108/20 | T2-L5 | PSO |
| 7 | F | 38 | 26.1 | IS | 97.5/T4-L1/T8 | 24.7 | 15.6 | -21.5 | -17.4 | 72/25 | T2-L4 | PSO |
| 8 | F | 26 | 17.5 | IS | 92.7/T6-T12/T89 | 22.8 | -1.2 | 105.4 | 4.3 | 68/20 | T3-L4 |  |
| 9 | F | 20 | 14.8 | IS | 105.3/T7-L2/T10 | 7.6 | 11.8 | 57.4 | -16.5 | 116/20 | T2-L4 | SPO, VCR |
| 10 | F | 32 | 15.9 | IS | 91.3/T3-L1/T9 | 15.3 | 3.3 | 61.8 | -2.9 | 68/17.5 | T2-L4 | PSO |
| 11 | F | 27 | 21.3 | IS | 118.7/T4-L2/T10 | 6.3 | 16.5 | 57.1 | 11.0 | 105/25 | T2-L5 | VCR |
| 12 | F | 41 | 16.8 | IS | 93.9/T4-L1/T9 | 22.5 | -1.4 | 32.6 | 4.7 | 49/20 | T2-L4 | PSO |
| 13 | F | 33 | 20.8 | IS | 96.8/T6-L1/T9 | 16.3 | 26.8 | 72.8 | 9.4 | 82/25 | T2-L3 |  |
| 14 | F | 26 | 17.9 | IS | 90.3/T5-L1/T10 | 20.5 | 7.9 | 9.4 | 29.3 | 33/17.5 | T3-L5 | PSO |
| 15 | F | 33 | 16.2 | IS | 92.3/T3-L2/T9 | 14.7 | -22.4 | 38.6 | -23.6 | 59/15 | T2-L4 |  |
| 16 | F | 30 | 22.6 | IS | 95.5/T4-T12/T8 | 6.2 | -37.6 | 70.6 | 35.4 | 73/25 | T1-L3 | PSO |
| 17 | F | 34 | 24.6 | IS | 115.3/T4-L1/T89 | 2.8 | 1.9 | 35.2 | -3.6 | 70/25 | T2-L4 | VCR |
| 18 | M | 28 | 20.8 | IS | 91.6/T6-L1/T9 | 12.9 | -17.8 | 92.5 | -17.5 | 68/25 | T2-L4 |  |
| 19 | M | 27 | 22.1 | IS | 102.4/T6-T12/T9 | 11.1 | 18.8 | 73.9 | 30.8 | 107/25 | T2-L4 | PSO |
| 20 | M | 19 | 24.5 | IS | 93.6/T6-L2/T9 | 6.7 | 3.7 | -9.8 | 28.4 | 103/25 | T2-L4 | PSO |
| 21 | M | 25 | 21.9 | IS | 95.7/T4-L1/T89 | 10.9 | 11.8 | 37.1 | -30.9 | 73/25 | T2-L4 | PSO |
| 22 | M | 30 | 18.3 | IS | 91.4/T5-L2/T10 | 17.2 | 27.2 | -11.6 | -3.7 | 47/15 | T3-L4 |  |
| 23 | F | 37 | 14.6 | NM | 96.5/T4-L3/T9 | 8.8 | -39.8 | 69.3 | -12.5 | 94/10 | T2-L4 | PSO |
| 24 | F | 34 | 24.3 | NM | 96.2/T4-L1/T8 | 22.3 | 31.4 | 89.7 | 39.2 | 76/25 | T1-L4 | PSO |
| 25 | F | 27 | 19.6 | NM | 93.9/T6-L2/T10 | 15.4 | 24.7 | -14.4 | 33.5 | 42/20 | T2-L5 | SPO, VCR |
| 26 | F | 32 | 15.3 | NM | 96.4/T4-L2/T9 | 13.2 | -12.6 | 16.2 | -10.4 | 54/15 | T2-L4 | VCR |
| 27 | F | 28 | 21.2 | NM | 92.3/T5-L1/T9 | 20.4 | -33.2 | 35.8 | 9.0 | 61/20 | T2-L4 |  |
| 28 | F | 19 | 14.7 | NM | 99.6/T4-L1/T8 | 5.2 | 8.6 | 97.1 | -26.6 | 114/15 | T2-L4 | SPO, PSO |
| 29 | F | 40 | 16.4 | NM | 93.7/T5-L2/T9 | 1.9 | 26.0 | 77.3 | -8.4 | 125/15 | T2-L4 | SPO, PSO |
| 30 | F | 38 | 28.4 | NM | 96.3/T4-L1/T9 | 12.3 | -10.8 | 41.0 | 13.1 | 55/25 | T2-L4 | SPO, VCR |
| 31 | M | 29 | 19.6 | NM | 124.5/T5-L2/T11 | 2.9 | 12.6 | 106.2 | 27.5 | 102/25 | T2-L4 | VCR |
| 32 | M | 36 | 23.7 | NM | 98.7/T6-L1/T8 | 9.4 | -8.5 | -17.3 | -16.2 | 59/25 | T2-L4 | SPO, PSO |
| 33 | M | 20 | 21.5 | NM | 95.3/T7-L1/T8 | 15.1 | 36.2 | -3.9 | 27.2 | 42/25 | T2-L3 | VCR |
| 34 | M | 26 | 16.9 | NM | 113.4/T6-L1/T10 | 10.9 | 25.4 | 62.8 | -19.6 | 98/20 | T3-L3 | SPO, PSO |
| 35 | M | 30 | 15.7 | NM | 92.8/T4-L1/T89 | 11.4 | 35.5 | 58.5 | 22.7 | 47/17.5 | T2-L4 | PSO |
| 36 | F | 21 | 21.6 | CS | 123.3/T4-T11/T78 | 8.2 | 38.6 | 102.7 | -32.3 | 97/20 | T2-L4 | SPO, VCR |
| 37 | F | 27 | 27.9 | CS | 91.1/T3-L2/T9 | 7.9 | -21.4 | 49.7 | 26.2 | 109/25 | T1-L4 | PSO |
| 38 | F | 34 | 23.4 | CS | 91.6/T6-L2/T10 | 7.2 | -24.7 | 36.2 | 25.1 | 69/25 | T2-L4 | PSO |
| 39 | F | 26 | 26.6 | CS | 117.7/T4-T12/T8 | 13.6 | -19.3 | 97.9 | 13.6 | 52/25 | T2-L3 | PSO |
| 40 | F | 32 | 14.4 | CS | 92.6/T5-L2/T10 | 10.8 | 2.6 | -21.8 | -17.4 | 86/15 | T2-L4 | SPO, VCR |
| 41 | F | 24 | 19.2 | CS | 93.5/T3-L1/T10 | 15.4 | -4.7 | -10.4 | 25.1 | 47/20 | T2-L4 | SPO, PSO |
| 42 | M | 26 | 18.1 | CS | 101.5/T5-L3/T9 | 11.8 | 24.5 | 83.6 | -12.6 | 63/25 | T2-L4 | SPO, PSO |
| 43 | M | 35 | 22.6 | CS | 95.8/T4-L2/T89 | 14.3 | 14.8 | 81.6 | 30.2 | 106/25 | T2-L4 | VCR |
| 44 | M | 33 | 15.3 | CS | 92.7/T4-L2/T8 | 18.8 | 4.4 | -10.4 | 4.1 | 88/17.5 | T2-L4 | SPO, PSO |
| 45 | M | 31 | 22.9 | CS | 104.3/T4-L1/T10 | 9.3 | 34.6 | 77.4 | -3.8 | 102/25 | T2-L4 | VCR |
| 46 | M | 39 | 23.7 | CS | 94.3/T6-L2/T10 | 8.4 | -19.9 | 53.5 | 17.6 | 42/25 | T2-L4 | PSO |
| 47 | M | 28 | 17.1 | CS | 91.6/T3-T12/T7 | 7.6 | 12.3 | 76.3 | -20.8 | 42/20 | T2-L3 | PSO |

IS idiopathic scoliosis; NM neuromuscular; CS congenital scoliosis; BMI body mass index; PSO pedicle subtraction osteotomy; SPO Smith-Petersen osteotomy; VCR vertebral column resection

*Flexibility index (%) = (preoperative Cobb angle − preoperative side bending Cobb angle)/preoperative Cobb angle (%).
