# Supplementary material for: Pulmonary Recovery Following Corrective Surgery in Adult Patients With Severe Scoliosis: A Minimum of Five-Year Follow-Up
Source: Front Med (Lausanne). 2022 Jun 16;9:915904. doi: 10.3389/fmed.2022.915904 (PMC9243643; doi:10.3389/fmed.2022.915904)
Supplement: Supplementary file 2 [file Table_2.DOCX]

| Supplementary table 2. Spirometric measurements and lung volume results in patients with different etiology of scoliosis | | | | |
| --- | --- | --- | --- | --- |
| Outcome | Idiopathic scoliosis (n=22) | Congenital scoliosis (n=12) | Neuromuscular scoliosis (n=13) | P value |
| FVC prior to traction | 2.19(0.79) | 2.20(0.99) | 2.62(0.60) | 0.43 |
| FVC at postoperative 5-year follow-up | 2.28(0.66) | 2.45(1.13) | 2.69(0.67) | 0.54 |
| Change in FVC | 0.09(0.65) | 0.25(0.48) | 0.07(0.85) | 0.63 |
| FVC% prior to traction | 61.5(27.8) | 62.2(24.5) | 64.4(22.7) | 0.87 |
| FVC% at postoperative 5-year follow-up | 62.6(24.6) | 64.1(23.2) | 67.3(23.8) | 0.82 |
| Change in FVC% | 1.1(17.9) | 1.9(20.1) | 2.9(19.4) | 0.57 |
| FEV1 prior to traction | 1.93(0.76) | 1.95(0.43) | 2.05(1.02) | 0.94 |
| FEV1 at postoperative 5-year follow-up | 2.13(0.61) | 2.19(0.77) | 2.60(0.71) | 0.36 |
| Change in FEV1 | 0.20(0.58) | 0.24(0.82) | 2.55(0.88) | 0.62 |
| FEV1% prior to traction | 63.2(28.9) | 65.1(21.4) | 68.2(22.5) | 0.70 |
| FEV1% at postoperative 5-year follow-up | 67.8(23.6) | 69.6(25.3) | 73.0(25.7) | 0.65 |
| Change in FEV1% | 4.5(13.2) | 4.5(15.7) | 4.7(17.6) | 0.88 |
| Total lung volume prior to traction | 893.7(34.1) | 875.2(27.0) | 891.9(31.6) | 0.73 |
| Total lung volume at postoperative 5-year follow-up | 1137.6(36.2) | 998.7(34.9) | 1116.9(27.3) | 0.63 |
| Change in total lung volume | 242.8(41.5) | 123.4(38.6) | 223.9(32.8) | 0.08 |

*FEV1* forced expiratory volume in 1s, *FEV1%* the actually measured value/predicted value of FEV1, *FVC* forced vital capacity, *FVC %* the actually measured value/predicted value of FVC

Values are expressed as mean (SD)
